# Supplementary material for: Mitochondria supply ATP to the ER through a mechanism antagonized by cytosolic Ca2+
Source: eLife. 2019 Sep 9;8:e49682. doi: 10.7554/eLife.49682 (PMC6763289; doi:10.7554/eLife.49682)
Supplement: Supplementary file 1. [file elife-49682-supp1.doc]

Mitochondria supply ATP to the ER through a mechanism antagonized by cytosolic Ca^2+^

Jing Yong, *et al.*

**Supplementary file 1**

Summary of reporters used in CHO cells and their intended specificity.

| **Name** | **Reporter for** | **Compartment Localization** | **Imaging property** | **Reference** |
| --- | --- | --- | --- | --- |
| *mtAT 1.03* | ATP level | Mito matrix | FRET | *Imamura et al.,* 2009 |
| *ERAT 4.01 N7Q* | ATP level | ER lumen | FRET | *Vishnu et al.,* 2014 |
| *TagRFP* | Cytosolic location | Cytosol | Fluorescent | *Merzlyak et al.*, 2007 |
| *Perceval HR* | ATP/ADP ratio | Cytosol | Ratiometric | *Tantama et al.*, 2013 |
| *D1ER* | Ca^2+^ level | ER lumen | FRET | *Palmer et al.*, 2004 |
| *GEM-CEPIA1er* | Ca^2+^ level | ER lumen | Ratiometric | *Suzuki et al.*, 2014 |
| *mtGEM-GECO1* | Ca^2+^ level | Mito matrix | Ratiometric | *Zhao et al*., 2011 |
| *ER-RFP* | ER localization | ER lumen | Fluorescent | *Snapp et al.*, 2006 |
| *cpYFP* | pH level | Cytosol | Ratiometric | *Zhao et al.*, 2015 |
