# Supplementary material for: Mitochondria supply ATP to the ER through a mechanism antagonized by cytosolic Ca2+
Source: eLife. 2019 Sep 9;8:e49682. doi: 10.7554/eLife.49682 (PMC6763289; doi:10.7554/eLife.49682)
Supplement: Supplementary file 2. [file elife-49682-supp2.doc]

Mitochondria supply ATP to the ER through a mechanism antagonized by cytosolic Ca^2+^

Jing Yong, *et al.*

**Supplementary file 2**

**Free Ca^2+^ concentration estimates for CaCl_2_ containing respiration buffers**

| Other chelating ingredients | CaCl_2_ (mM) | Ca^2+^ (nM) |
| --- | --- | --- |
| Ionic strength = 0.029 N,  and  EGTA 1 mM  Mg^2+^ 5 mM  ATP = ~ 0.2 mM  pH ~7.4, at 25ºC | 0.100 | 12.9 |
|  | 0.250 | 40.2 |
|  | 0.500 | 118 |
|  | 0.750 | 357 |
|  | 0.800 | 478 |
|  | 0.875 | 840 |
|  | 0.900 | 1,000 |
|  | 0.950 | 2,100 |

**Tool used for estimate:** https://somapp.ucdmc.ucdavis.edu/pharmacology/bers/maxchelator/CaMgATPEGTA-TS-Plot.htm
