# Supplementary material for: Mitochondria supply ATP to the ER through a mechanism antagonized by cytosolic Ca2+
Source: eLife. 2019 Sep 9;8:e49682. doi: 10.7554/eLife.49682 (PMC6763289; doi:10.7554/eLife.49682)
Supplement: Transparent reporting form [file elife-49682-transrepform.docx]

***eLife’s* transparent reporting form**

We encourage authors to provide detailed information *within their submission* to facilitate the interpretation and replication of experiments. Authors can upload supporting documentation to indicate the use of appropriate reporting guidelines for health-related research (see [EQUATOR Network](http://www.equator-network.org/%20)), life science research (see the [BioSharing Information Resource](https://biosharing.org/)), or the [ARRIVE guidelines](http://www.plosbiology.org/article/info:doi/10.1371/journal.pbio.1000412) for reporting work involving animal research. Where applicable, authors should refer to any relevant reporting standards documents in this form.

If you have any questions, please consult our Journal Policies and/or contact us: [editorial@elifesciences.org](mailto:editorial@elifesciences.org).

**Sample-size estimation**

- You should state whether an appropriate sample size was computed when the study was being designed
- You should state the statistical method of sample size computation and any required assumptions
- If no explicit power analysis was used, you should describe how you decided what sample (replicate) size (number) to use

**Sample-size estimation** –

Since we used both flow cytometry based FRET assay and fluorescent microscope based FRET assay, the sample-size estimation is contingent on the method.

Specifically, for flow cytometry based FRET assay, sample size is > 3000 individual cells per data point. And we observed that the average and the distribution of the ATP signal stabilize after a sample size of ~ 1000 cells during the analysis.

For fluorescent microscopy-based FRET measurement, we observed that the signal stabilizes after the averages from N>=3 independent replicate experiments were combined for every condition, with multiple cells (n >=2) for each replicate. A typical result is shown in the main figures (e.g. **Fig. 2C** and **2D**), which as an example includes a combination of >10 cells per group, with individual cell traces represented in grey solid lines, or in dots (e.g. Fig. 4K).

Please outline where this information can be found within the submission (e.g., sections or figure legends), or explain why this information doesn’t apply to your submission:

**Replicates**

- You should report how often each experiment was performed
- You should include a definition of biological versus technical replication
- The data obtained should be provided and sufficient information should be provided to indicate the number of independent biological and/or technical replicates
- If you encountered any outliers, you should describe how these were handled
- Criteria for exclusion/inclusion of data should be clearly stated
- High-throughput sequence data should be uploaded before submission, with a private link for reviewers provided (these are available from both GEO and ArrayExpress)

Please outline where this information can be found within the submission (e.g., sections or figure legends), or explain why this information doesn’t apply to your submission:

Replicates –

Each experiment has been replicated technically for more than three times, and the main conclusions were further tested with different compounds exerting similar effects, e.g. for ***Fig. 1***, we tested three OxPhos inhibitors – oligomycin, rotenone, and FCCP; for ***Fig. 2*** and ***3***, we tested three SERCA inhibitors – BHQ, thapsigargin, and cyclopiazonic acid.

To ensure that the *CaATiER* phenomenon is not cell-type specific, we further tested three cell lines, i.e. Chinese Hamster Ovary (CHO) cell, rat insulinoma INS-1 cell, and human cervical cancer cell HeLa cell. CaATiER phenomenon is highly reproducible in all the cell types we tested.

**Statistical reporting**

- Statistical analysis methods should be described and justified
- Raw data should be presented in figures whenever informative to do so (typically when N per group is less than 10)
- For each experiment, you should identify the statistical tests used, exact values of N, definitions of center, methods of multiple test correction, and dispersion and precision measures (e.g., mean, median, SD, SEM, confidence intervals; and, for the major substantive results, a measure of effect size (e.g., Pearson's r, Cohen's d)
- Report exact p-values wherever possible alongside the summary statistics and 95% confidence intervals. These should be reported for all key questions and not only when the p-value is less than 0.05.

Please outline where this information can be found within the submission (e.g., sections or figure legends), or explain why this information doesn’t apply to your submission:

**Statistical reporting**

A detailed description of statistical method used can be found in ***Supplemental Information***, Section 5 – under “*Quantification and Statistical Analysis*”. Since we used both flow cytometry based FRET assay and fluorescent microscope based FRET assay, the statistical analysis is contingent on the method.

For flow cytometry based FRET assay the geometric means of fluorescence intensities (gMFI) were applied for population statistics analysis, as ratiometric parameters usually follow a log-normal distribution. Compartmental ATP and/or Ca2+ levels were compared using the “*Ordinary Two-way ANOVA*” function provided by GraphPad’s *Prism* software (Ver: 7.0), with alpha level of 0.05. Time and compounds were assumed two independent parameters for *ANOVA* analysis. *Dunnett*’s multiple comparisons test was subsequently performed for comparison between individual groups versus diluent control (DMSO or PBS), or control group as indicated for particular experiments.

Secondly for flow cytometry based FRET assay - Since large number of cells were analyzed for every data point, plotting error bars of SEM was often difficult, as **SEM** = Standard deviation (S.D.)/ Square root of (N), where N = sample size. We deem it obvious that a large N will inevitably reduce the absolute value of the SEM, making it difficult to add an error bar, as “*For some points, the error bars would be shorter than the height of the symbol. In these cases, Prism simply does not draw the error bars.*” (Quoting from the Prism statistical analysis software)

For fluorescent microscopy-based FRET measurement, statistical methods used are: 1). *Student*’s t-test (unpaired), for comparison of two variables from independent measurements (e.g. **Fig. 4K**); 2). One-way *ANOVA*, for comparison of multiple data-points or variables (>2) from independent measurements (e.g. **Fig 1** - *Figure Supplement 5G* and **Fig. 2** – *Figure Supplement 1B and 1C*).

(For large datasets, or papers with a very large number of statistical tests, you may upload a single table file with tests, Ns, etc., with reference to sections in the manuscript.)

**Group allocation**

- Indicate how samples were allocated into experimental groups (in the case of clinical studies, please specify allocation to treatment method); if randomization was used, please also state if restricted randomization was applied
- Indicate if masking was used during group allocation, data collection and/or data analysis

Cells were randomly chosen to be allocated to experimental groups.

For comparison of compound-exerted ER ATP effect by flow cytometry based FRET assay, cells were prepared and allocated using the following scheme –

Please outline where this information can be found within the submission (e.g., sections or figure legends), or explain why this information doesn’t apply to your submission:

**Additional data files (“source data”)**

- We encourage you to upload relevant additional data files, such as numerical data that are represented as a graph in a figure, or as a summary table
- Where provided, these should be in the most useful format, and they can be uploaded as “Source data” files linked to a main figure or table
- Include model definition files including the full list of parameters used
- Include code used for data analysis (e.g., R, MatLab)
- Avoid stating that data files are “available upon request”

Please indicate the figures or tables for which source data files have been provided:

Source data files are curated and stored in *.xls* format, *.fcs* format and in *.pzf* format, all of which are available upon request.
